# Interacting host modifier systems control *Wolbachia*‐induced cytoplasmic incompatibility in a haplodiploid mite

**DOI:** 10.1002/evl3.282

**Published:** 2022-05-11

**Authors:** Nicky Wybouw, Frederik Mortier, Dries Bonte

**Affiliations:** ^1^ Terrestrial Ecology Unit, Department of Biology Faculty of Sciences, Ghent University Ghent Belgium

**Keywords:** Genetic conflict, host modification, pest control, reproductive parasitism

## Abstract

Reproductive parasites such as *Wolbachia* spread within host populations by inducing cytoplasmic incompatibility (CI). CI occurs when parasite‐modified sperm fertilizes uninfected eggs and is typified by great variation in strength across biological systems. In haplodiploid hosts, CI has different phenotypic outcomes depending on whether the fertilized eggs die or develop into males. Genetic conflict theories predict the evolution of host modulation of CI, which in turn influences the stability of reproductive parasitism. However, despite the ubiquity of CI‐inducing parasites in nature, there is scarce evidence for intraspecific host modulation of CI strength and phenotype. Here, we tested for intraspecific host modulation of *Wolbachia*‐induced CI in haplodiploid *Tetranychus urticae* mites. Using a single CI‐inducing *Wolbachia* variant and mitochondrion, a nuclear panel was created that consisted of infected and cured near‐isogenic lines. We performed a highly replicated age‐synchronized full diallel cross composed of incompatible and compatible control crosses. We uncovered host modifier systems that cause striking variation in CI strength when carried by infected *T. urticae* males. We observed a continuum of CI phenotypes in our crosses and identified strong intraspecific female modulation of the CI phenotype. Crosses established a recessive genetic basis for the maternal effect and were consistent with polygenic Mendelian inheritance. Both male and female modulation interacted with the genotype of the mating partner. Our findings identify spermatogenesis as an important target of selection for host modulation of CI strength and underscore the importance of maternal genetic effects for the CI phenotype. Our findings reveal that intraspecific host modulation of CI is underpinned by complex genetic architectures and confirm that the evolution of reproductive parasitism is contingent on host genetics.

**Impact Summary**

Maternally inherited bacteria infect most arthropod species and can spread within host populations by manipulating host reproduction. Cytoplasmic incompatibility (CI) is the most common reproductive manipulation and is mainly associated with *Wolbachia* bacteria. CI occurs when infected males mate with uninfected females, providing a selective advantage to infected females that transmit *Wolbachia. Wolbachia*‐induced CI is typified by great phenotypic variation across biological systems. Although predicted by theory, whether and to what extent arthropod hosts modulate CI remain poorly understood. Here, we tested for intraspecific host modulation of *Wolbachia*‐induced CI in *Tetranychus urticae* spider mites. We transferred a single CI‐inducing *Wolbachia* isolate into multiple host nuclear backgrounds. Using this nuclear panel, the contribution of the host genotype to CI was quantified and dissected. We observed striking variation in CI, underscoring the importance of host genetics for *Wolbachia‐*host interactions. We uncovered that infected males modulate different features of CI compared to uninfected females. Interestingly, both male and female modulation interacted with the genotype of the mating partner, revealing that these mechanisms are underpinned by complex genetic architectures. Our findings help elucidate the mechanisms that underlie reproductive parasitism.

Endosymbiotic reproductive parasites facilitate their maternal transmission by manipulating host reproduction (Engelstädter and Hurst 2009; Perlmutter and Bordenstein 2020). Cytoplasmic incompatibility (CI) is the most common reproductive manipulation and induces defects in paternal chromosome condensation and segregation in developing embryos when infected males mate with uninfected females (Shropshire et al. 2020). In diploid hosts, these defects in paternal chromosome behavior result in embryonic mortality, whereas in hosts with a haplodiploid mode of reproduction (unfertilized haploid eggs develop into males, fertilized diploid eggs result in females), two different CI phenotypes can be distinguished. Chromosomal defects can give rise to viable haploid male offspring and are commonly referred to as Male Development CI (MD‐CI) (Vavre et al. 2001; Perrot‐Minnot et al. 2002). Alternatively, CI in haplodiploids can lead to aneuploidy in fertilized eggs and induce female mortality in incompatible crosses (Female Mortality CI, FM‐CI) (Breeuwer 1997; Vavre et al. 2001; Perrot‐Minnot et al. 2002). Reproductive parasites can also induce a mix of both FM‐CI and MD‐CI in haplodiploid arthropods, suggesting that the phenotypic outcomes of incompatible crosses can lie along a continuum with FM‐CI and MD‐CI as the extremes (Breeuwer 1997; Vavre et al. 2001, 2000; Perrot‐Minnot et al. 2002; Bordenstein et al. 2003; Nguyen et al. 2017). CI is ablated when infected males mate with infected females and therefore provides a selective advantage to females that transmit the reproductive parasite.

CI is primarily associated with *Wolbachia*, but other bacteria can also induce CI in their arthropod host (Shropshire et al. 2020). The genetic architecture of *Wolbachia*‐mediated CI is a pair of syntenic genes (*cifA* and *cifB*) that are located in WO prophage regions of certain *Wolbachia* genomes (Beckmann et al. 2019a, 2017; LePage et al. 2017). Across different host systems, CI strength is highly variable and is typically associated with varying *Wolbachia* frequencies within host populations (Hoffmann et al. 1990). Although previous work has shown that natural genetic variation of *cif* operons can influence CI strength (Martinez et al. 2020; Beckmann et al. 2021; Shropshire et al. 2021), *Wolbachia* genetic diversity does not sufficiently explain CI strength and phenotype variation. Host background can be a strong determining factor for CI strength and phenotype, an observation that is primarily supported by interspecific transfer of *Wolbachia* and host species hybridization (Holden et al. 1993; Poinsot et al. 1998; Reynolds and Hoffmann 2002; Bordenstein et al. 2003; Sakamoto et al. 2005; Zabalou et al. 2008; Walker et al. 2011).

Interspecific modulation of CI is consistent with genetic conflict theories that predict the evolution of host modifier systems in populations that are polymorphic for *Wolbachia* infection (Turelli 1994). However, intraspecific modulation of CI is not well documented, nor understood, in part due to variation caused by (asymmetrical) nuclear‐ or mitochondrial‐associated incompatibilities (Hoffmann and Turelli 1988; Reynolds and Hoffmann 2002; Carrington et al. 2011; Cooper et al. 2017). Intraspecific variation in CI strength and phenotype has been observed for the haplodiploid spider mite *Tetranychus urticae* and the parasitoid wasp *Leptopilina heterotoma* (Breeuwer 1997; Vavre et al. 2001; Perrot‐Minnot et al. 2002; Mouton et al. 2005), raising questions about the ability of haplodiploid hosts to control the different features of CI. The mechanistic underpinnings of intraspecific host modulation are not known. Host modulation mechanisms can be expressed in males, females, and embryos of incompatible crosses. In males, these mechanisms limit *Wolbachia*‐induced aberrations of spermatogenesis to produce normal, healthy sperm. Females can modify CI through maternal effects that operate during oogenesis and embryogenesis. For instance, maternally supplied histones are vital for the formation of the male pronucleus and display delayed deposition in embryos from incompatible crosses, findings that identify this process as a potential target for host selection (Loppin et al. 2005; Landmann et al. 2009). Finally, the embryo can also modulate the effects of CI throughout the early and later stages of its development (LePage et al. 2017; Nguyen et al. 2017; Shropshire et al. 2020). Whether host modifier systems control different features of CI or combine additively or synergistically is unknown, raising pertinent questions for our understanding of reproductive parasitism and the development of effective CI‐based pest management. Indeed, infected males are released into genetically diverse pest populations and reduce population growth by inducing strong CI. Segregating modifier systems in wild pest populations can severely threaten the long‐term stability and effectiveness of CI‐based pest control (Ross et al. 2019; Utarini et al. 2021).

To study the mechanistic underpinnings of host modulation in a haplodiploid arthropod, we created a nuclear panel of *T. urticae* comprising different nuclear backgrounds, a single mitochondrion, and a single CI‐inducing *Wolbachia* variant. We performed a highly replicated full diallel cross and quantified variation in CI strength and phenotype using Bayesian inference and corrected indexes that control for nuclear and temporal effects. CI strength varied from very weak to complete and was strongly determined by the male genotype. CI phenotypes ranged along a continuum and were determined by the female and male genotype as well as their interaction. Genetic crosses uncovered insights into the genetic architecture underlying female modulation of the CI phenotype. Together, our findings reveal multiple mechanisms of host modulation of CI within a single haplodiploid arthropod species.

## Materials and Methods

### CREATION AND CHARACTERIZATION OF THE *Tetranychus* NUCLEAR PANEL

**Figure 1:**
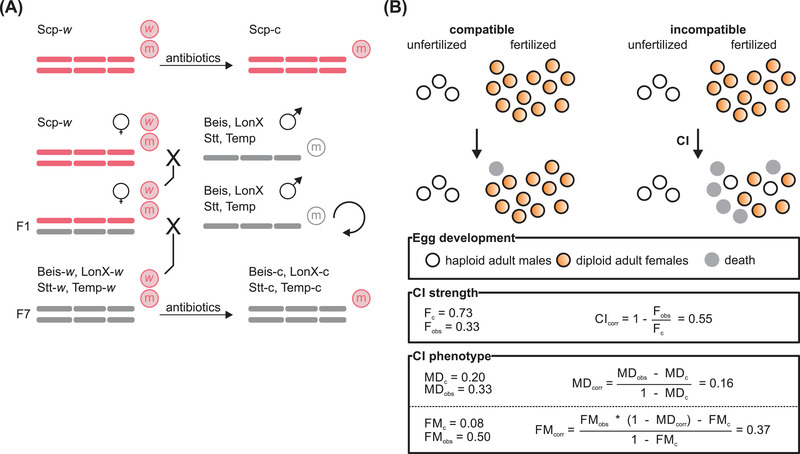
The creation of a *T. urticae* nuclear panel and the use of corrected metrics for the CI features control for nuclear‐ and mitochondrial‐associated incompatibilities. (A) The experimental design that created the nuclear panel. *Wolbachia* was transferred from Scp‐*w* into the Beis, LonX, Stt, and Temp nuclear backgrounds by paternal introgression, creating Beis‐*w*, LonX‐*w*, Stt‐*w*, and Temp‐*w*. Infected lines were cured of *Wolbachia* by antibiotic treatment, creating Beis‐c, LonX‐c, Scp‐c, Stt‐c, and Temp‐c. The single *Wolbachia* variant and mitochondrion of Scp‐*w* are indicated by encircled ‘*w*’ and ‘m’ symbols in red font. (B) An informative example of a compatible and incompatible cross of a haplodiploid arthropod to illustrate the CI features and their metrics. Spheres indicate eggs and are color coded depending on their outcome. Using the proportion of adult female offspring over the total number of eggs for compatible and incompatible crosses (F_c_ and F_obs_), CI_corr_ estimates CI strength. MD_corr_ and FM_corr_ quantify the CI phenotype based on the proportion of adult male offspring over the total number of eggs (MD_c_ and MD_obs_ for compatible and incompatible crosses) and female mortality (FM_c_ and FM_obs_ for compatible and incompatible crosses), respectively. In the depicted incompatible cross, five fertilized eggs died, whereas two fertilized eggs developed into adult males. This is reflected in the FM_corr_ and MD_corr_ metrics.

A single teleiochrysalid (virgin) female was collected from four *T. urticae* field populations (Beis, Scp‐*w*, Stt, and Temp) and from a laboratory population (LonX) derived from the London reference strain (Grbić et al. 2011) (Table S1). To ensure near‐isogenic nuclear backgrounds, lines were created by three sequential rounds of mother‐son crosses and were finally founded by 20 adult females. After an oviposition period of three days, genomic DNA was extracted from these founding females with a Quick‐DNA Universal kit (BaseClear, the Netherlands). A fragment of the mitochondrial *COI* gene was sequenced for the Scp‐*w* line (Sanger sequencing, MACROGEN Europe B.V.) (Table S2). Infection of the five *Tetranychus* lines with the reproductive manipulators *Wolbachia*, *Rickettsia*, *Cardinium*, and *Spiroplasma* was tested using diagnostic PCR assays. PCR conditions are described in Table S2. The diagnostic PCR assays showed that none of the lines carried *Rickettsia*, *Cardinium*, or *Spiroplasma* and that only Scp‐*w* was infected with *Wolbachia*. The *Wolbachia* variant of Scp‐*w* was further characterized by multilocus sequence typing and by sequencing an ∼1,000 bp genomic fragment that brackets the *wsp* gene (Sanger sequencing, MACROGEN Europe B.V.) (Table S2) (Baldo et al. 2006). We transferred the *Wolbachia* variant into four other near‐isogenic backgrounds by paternal introgression, creating Beis‐*w*, LonX‐*w*, Stt‐*w*, and Temp‐*w*. For each near‐isogenic nuclear background, 20 *Wolbachia*‐infected Scp‐*w* females were crossed to 15 uninfected males, and 25 infected female offspring were backcrossed to 15 males of the uninfected genotype for an additional six generations (Figure 1A). The fidelity of *Wolbachia* maternal transmission was tested in the five infected nuclear backgrounds. DNA was extracted from individual adults (electronic supplementary material), and the diagnostic PCR assays were performed as described above (Table S2). The total numbers of tested mites are listed in Table S3. The Beis‐*w*, LonX‐*w*, Scp‐*w*, Stt‐*w*, and Temp‐*w* lines were cured of *Wolbachia* infection by antibiotic treatment (electronic supplementary material). After antibiotic curing, the cured lines Beis‐c, LonX‐c, Scp‐c, Stt‐c, and Temp‐c were maintained on detached bean leaves for at least four generations before *Wolbachia*‐mediated incompatibilities were phenotyped. The 10 lines of the mite panel were maintained by serial passage on detached bean leaves at a census size of ∼250 mites at 24°C, 60% RH, and a 16:8 light:dark photoperiod.

### HOST MODULATION OF *Wolbachia*‐INDUCED CI

Host modifier systems were identified and characterized in our *T. urticae* panel by performing incompatible (uninfected females to infected males) and compatible (uninfected females to uninfected males) intraspecific crosses in a full factorial diallel cross design. This experimental design generated a total of 50 cross types: 25 incompatible and 25 compatible control cross types. A second set of crosses tested the ability of infected females of Scp‐*w* and Beis‐*w* to rescue CI and consisted of rescue (infected females to infected males) and control (infected females to uninfected males) crosses. Age cohorts were created by allowing 50 mated females to oviposit for 24 hours on detached bean leaves. Each cross type consisted of eight to eleven replicates except for the rescue and respective control crosses, where five replicates were established per cross type (electronic supplementary material, Table S4). For each replicate, five female teleiochrysalids were paired with four one‐ to three‐day‐old adult males on a 16 cm^2^ leaf disc. After five days, the mites were discarded, and the eggs were counted. During development, adult male and female offspring were isolated and counted.

### GENETIC BASIS OF FEMALE MODULATION OF THE CI PHENOTYPE

We uncovered the mode of inheritance of female modulation of the CI phenotype using Scp‐c and LonX‐c females that express distinct CI phenotypes when crossed to Scp‐*w* males. Heterozygous F_1_ females were produced by reciprocal crosses between LonX‐c and Scp‐c. In each cross, 25 virgin females were paired with 20 adult males on a 16 cm^2^ leaf disc and allowed to mate and oviposit. During F_1_ development, female teleiochrysalids were isolated for the incompatible and compatible control crosses. Adult Scp‐*w* and Scp‐c males were obtained from synchronized age cohorts as previously described. Cross types consisted of seven to eight replicates, with five females and four males per replicate. After five days, the mites were discarded, and the eggs were counted. During development, adult male and female offspring were isolated and counted.

To obtain recombinant F_2_ females, 25 virgin F_1_ females were backcrossed to 20 LonX‐c males. Individual female F_2_ teleiochrysalids were isolated, paired with a single one‐ to three‐day‐old adult Scp‐*w* or Scp‐c male, and allowed to oviposit for seven days. Control cross types consisted of five to 21 replicates, whereas the incompatible cross types included 22 to 34 replicates. During development, adult male and female offspring were isolated and counted.

### STATISTICAL ANALYSIS OF CI STRENGTH AND PHENOTYPE

We modeled the proportion of adult female offspring over the total number of eggs (F), the proportion of adult male offspring over the total number of eggs (MD), and the proportion of eggs that failed to generate adult mites over the total number of eggs that did not generate adult males (FM). The numerators were modeled as responses with a binomial error distribution, while the denominators were modeled as numbers of draws. We estimated F, MD, and FM using full models. F, MD, and FM were modeled with effects from the *Wolbachia*‐infection state in males (*inf*), male genotype (*mgeno*), female genotype (*fgeno*), and all their interactions. The days at which the different cross types were initiated (*Day*) were included as variable intercepts. The full model for F can be formulated as follows:

F∼Binomial(p,eggs)

logit(p)=inf*mgeno*fgeno+(1|Day)

The full models for MD and FM were similarly built using the respective numerators and denominators. To study CI rescue, we estimated F using the full model that incorporates all effects and their interactions. To study the inheritance of female modulation, F, MD, and FM were modeled with the effects from the *Wolbachia*‐infection state in males and the female genotype. To test the hypothesis of a monogenic, recessive mode of inheritance, we modeled MD with an ordered mixture model of two binomial distributions. The estimated component weights of the mixture quantified the proportion of recombinant F_2_ females with an extreme MD phenotype (∼LonX‐c females). This proportion is expected to be 0.5 for a monogenic, recessive trait. For CI rescue and female modulation, the experimental design did not allow to estimate the variable day effect.

CI strength and phenotype were analyzed with Bayesian inference using the brms package (version 2.12.0) in R (version 3.6.3) (Bürkner 2018; R Core Team 2021). Statistical models were run using Hamiltonian Monte Carlo (HMC) that implemented two chains with each 5,000 iterations from which 1,000 were warmup (McElreath 2018). Priors were used that are weakly regularizing by choosing prior distributions that are significantly wider than the parameter values that would be reasonable to expect for each model. We evaluated model performance by checking mixing and stationarity in the trace plots and by checking the effective sample size and $\hat{R}$ statistic for each parameter (McElreath 2018). Models, formulae, and detailed descriptions are listed in the electronic supplementary material.

To control for variation in F, MD, and FM that is not related to *Wolbachia*‐induced CI (caused by nuclear and temporal effects), we used corrected indexes to quantify these CI features (Figure 1B). Using the model estimates of F, the corrected CI strength (CI_corr_) for each cross type was calculated from the posterior distribution of the full generalized linear model:

$$CI_{corr}=1-\frac{F_{obs}}{F_{c}}$$

where F_obs_ and F_c_ are the estimated F values in the incompatible and respective compatible crosses (Mouton et al. 2005; Nguyen et al. 2017). The corrected MD‐CI phenotype (MD_corr_) was calculated using the estimated MD values;

$$MD_{corr}=\frac{MD_{obs}-MD_{c}}{1-MD_{c}}$$

where MD_obs_ and MD_c_ are the estimated MD values in the incompatible and respective compatible crosses (Poinsot et al. 1998; Zélé et al. 2020; Cruz et al. 2021). The corrected FM‐CI phenotype (FM_corr_) was calculated using the estimated FM values;

$$FM_{corr}=\frac{FM_{obs}\;*\left(1-MD_{corr}\right)-FM_{c}}{1-FM_{c}}$$

where FM_obs_ and FM_c_ are the estimated FM values in the incompatible and respective compatible crosses (based on Poinsot et al. 1998; Zélé et al. 2020; Cruz et al. 2021).

To understand the importance of male and female genotypes and their interaction for intraspecific CI strength and phenotype, we compared four variations of the full models that consistently assumed an effect of the *Wolbachia*‐infection state in males but differed in the other fixed explanatory variables (male genotype, female genotype and their interactions) (electronic supplementary material). Models were compared using the Widely Applicable Information Criterion (WAIC). To quantify the relative impact of the different explanatory variables, we compared the finite‐population standard deviation of estimated coefficients for different explanatory variables and interactions in adjusted full models (electronic supplementary material).

## Results

We isolated a single CI‐inducing *Wolbachia* variant by generating a near‐isogenic line (Scp‐*w*) derived from a single *Wolbachia‐*infected field‐collected female (Figure S1). We created a *T. urticae* panel comprised of five near‐isogenic nuclear backgrounds that shared a single mitochondrion and were either infected with the CI‐inducing *Wolbachia* variant (Beis‐*w*, LonX‐*w*, Scp‐*w*, Stt‐*w*, and Temp‐*w*) or were cured of the infection (Beis‐c, LonX‐c, Scp‐c, Stt‐c, and Temp‐c). Complete maternal transmission of *Wolbachia* was observed in all five infected *T. urticae* nuclear backgrounds (Table S3). All compatible crosses produced F_1_ females, demonstrating fertilization across all near‐isogenic lines (electronic supplementary material).

### MALE MODIFIER SYSTEMS CAUSE CI STRENGTH VARIATION IN *Tetranychus*

**Figure 2:**
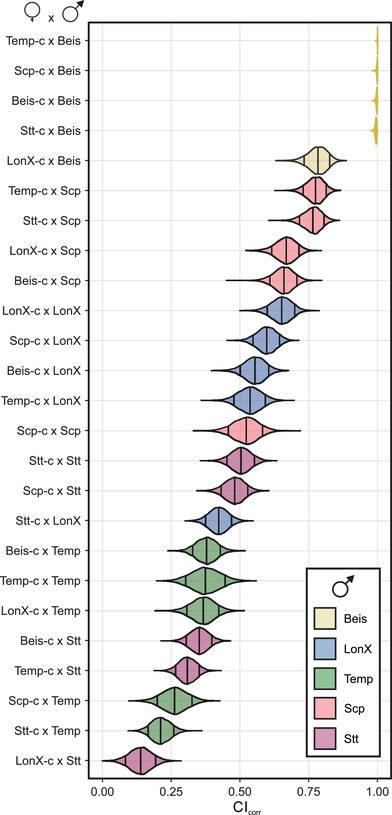
**Host modifier systems control CI strength in a *Tetranychus* spider mite**. Intraspecific CI strength variation within the full diallel cross design. Crosses are ordered according to decreasing CI strength. Violin plots are color coded based on the male genotype (see bottom right). CI strength was estimated using the CI_corr_ index, which controls for variation caused by nuclear and temporal effects (Figure 1). Each violin plot represents the estimated average for that cross and indicates the 0.09, 0.50 and 0.91 percentiles.

Using the model estimates of the full model (Figure S2), we calculated the corrected CI strength (CI_corr_) across the intraspecific cross types, controlling for nuclear and temporal effects (Figure 1B and 2). Intraspecific CI_corr_ varied greatly and ranged from complete to very weak. A model comparison was performed among models that differed in fixed explanatory variables to study the factors explaining intraspecific CI strength variation (Figure S3). These model comparisons revealed that the male genotype had the largest impact on model predictability, with its interaction with the female genotype as an important additional effect (Figure S3). Variance analysis confirmed that the male genotype greatly determined intraspecific CI strength with an additional substantial impact of the male–female genotype interaction (Figure S4). In contrast, the coefficients of all other model levels exhibited markedly lower levels of variation (Figure S4). These analyses indicate that (some) *T. urticae* male genotypes carry modifier systems that strongly modulate intraspecific CI strength.

*Wolbachia*‐infected Beis‐*w* males induced complete (or near‐complete) CI_corr_ when crossed to four uninfected female genotypes (Beis‐c, Scp‐c, Stt‐c, and Temp‐c). In contrast, all other genetic crosses revealed reduced levels of CI_corr_. The weakest CI_corr_ was observed in the crosses with infected Stt‐*w* and Temp‐*w* males, whereas infected Scp‐*w* and LonX‐*w* males induced intermediate levels of CI_corr_ (Figure 2). The level of interaction of the male and female genotypes on CI_corr_ varied across the intraspecific crosses (Figure 2 and Figure S5). The strongest interaction was observed when infected Beis‐*w* males were crossed with uninfected LonX‐c females, resulting in a CI_corr_ of ∼75% (Figure 2 and Figure S5). Together, these findings suggest that our *T. urticae* panel is typified by nuclear modifier systems of CI that are expressed in males and interact with the female genotype. The ability of infected females to rescue intraspecific CI was confirmed for the Scp and Beis genotypes using replicated age‐synchronized rescue (infected females to infected males) and control cross types (infected females to uninfected males) (Figure S6).

### MALE MODIFIER SYSTEMS CAUSE CI STRENGTH VARIATION BY CONTROLLING FEMALE MORTALITY

**Figure 3:**
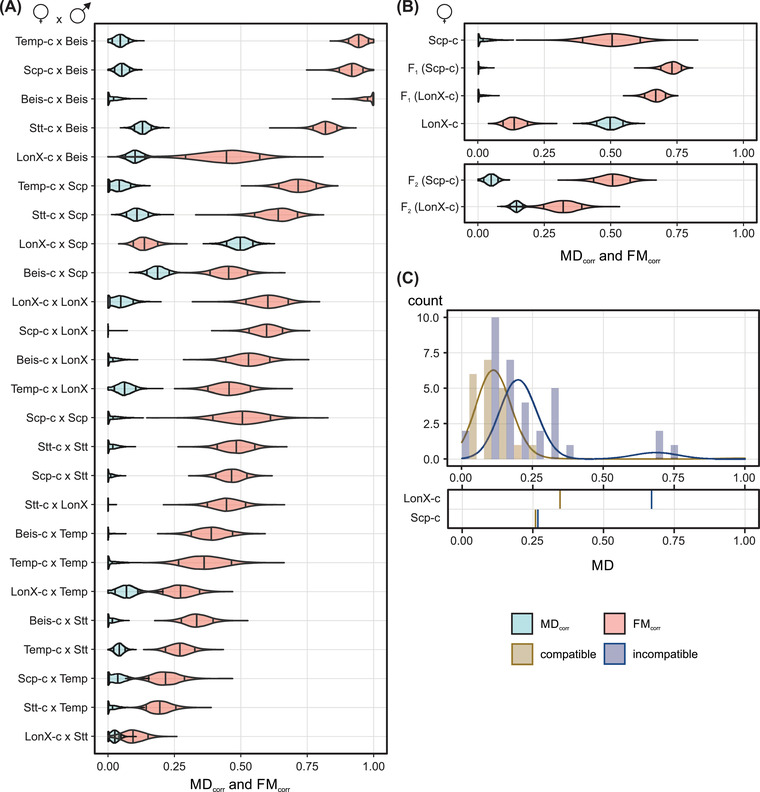
**Host modulation of the CI phenotype in a *Tetranychus* spider mite. (A)** Intraspecific CI phenotype variation within the full diallel cross design. Crosses are ordered according to decreasing CI strength. The CI phenotype was estimated using the MD_corr_ and FM_corr_ indexes that control for variation caused by nuclear and temporal effects (Figure 1). **(B)** Inheritance of the maternal genetic effect that contributes to intraspecific MD_corr_ variation. For the heterozygous F_1_ and recombinant F_2_ females, the genotype between brackets represents the original maternal genotype. All uninfected females were crossed to Scp‐*w* and Scp‐c males. The MD_corr_ and FM_corr_ estimates of LonX‐c and Scp‐c are identical to those of panel A. For both panels, violin plots of MD_corr_ and FM_corr_ display a blue and red background, respectively (see bottom right), represent the estimated averages for each cross, and indicate the 0.09, 0.50 and 0.91 percentiles. **(C)** Distribution of MD in the incompatible and compatible crosses of recombinant F_2_ females and Scp‐*w* and Scp‐c males. Cross compatibility is color coded (see bottom right). The average MD values for parental LonX‐c and Scp‐c are shown in the bottom plot. Bars represent the data distribution, and the density plots reflect the posterior prediction distributions of the mixture model.

The total CI strength in haplodiploids is the combined effect of MD‐CI and FM‐CI, where incompatibility is expressed as an excess of haploid male offspring or an increased mortality of female offspring, respectively. Using the model estimates of the full models, we calculated the corrected MD‐CI and FM‐CI indexes (MD_corr_ and FM_corr_, respectively) (Figure S7 and Figure S8). We observed a continuum for both CI phenotypes within *T. urticae*, but FM_corr_ variation was considerably larger (Figure 3A). The variation in MD_corr_ and FM_corr_ across the intraspecific crosses was examined by running several statistical models with the *Wolbachia* infection state in males as a consistent explanatory variable. Model comparisons for MD‐CI indicated that male and female genotypes and their interaction contributed equally to model predictability (Figure S9) but accounted for a relatively low amount of variation (Figure S10). In contrast, model comparisons and variance analysis revealed that the male genotype and, to a lesser extent, its interaction with the female genotype were important determinants for FM‐CI, showing a correlation between CI strength and FM‐CI in our data (Figure S9 and Figure S10). Correlation plots confirmed that FM_corr_ and CI_corr_ were tightly coupled, whereas patterns with MD_corr_ were inconsistent (Figure S11). Together, these findings suggest that the control of male modifier systems of intraspecific CI strength was mainly regulated by changes in the mortality rate of female offspring (Figure 3A).

### A RECESSIVE MATERNAL GENETIC EFFECT CONTRIBUTES TO INTRASPECIFIC MD‐CI VARIATION

The LonX‐c (♀) x Scp‐*w* (♂) cross was the only intraspecific incompatible cross where MD_corr_ (∼50%) exceeded FM_corr_ (∼12.5%) (Figure 3A and Figure S11). Crossing Scp‐*w* males to other uninfected female genotypes caused no or very weak intraspecific MD_corr_ (Figure 3A), suggesting that the relatively strong MD_corr_ resulted from an interaction between a maternal genetic feature of LonX with the Scp genotype. To gain further insight into female modulation of CI, we produced heterozygous F_1_ females by reciprocal crosses between LonX‐c and Scp‐c and performed replicated incompatible and compatible crosses with Scp males (Figure 3B). CI strength was stable between parents and F_1_ offspring with the incompatible crosses of both sets of F_1_ females and Scp‐*w* males, resulting in ∼70% CI_corr_ (Figure S12). Crossing heterozygous F_1_ females to Scp‐*w* males did not cause MD_corr_, establishing a recessive genetic basis for the LonX maternal effect (Figure 3B). We subsequently backcrossed F_1_ females to LonX‐c males and performed replicated incompatible and compatible crosses using a single recombinant F_2_ female per cross. The CI_corr_ remained stable and was ∼70% (Figure S12). We observed MD_corr_ values of ∼5% and ∼15% after crossing recombinant F_2_ females with Scp‐*w* (Figure 3B). We noted a clear change in the distribution of MD using recombinant F_2_ females with LonX‐c as the original maternal line (Figure 3C). After mating with Scp‐*w* males, an estimated 9% of recombinant F_2_ females produced a brood with an MD value that was similar to (or even exceeded) the average MD of LonX‐c (Figure 3C and Figure S13), patterns that are consistent with a polygenic basis.

## Discussion

Intraspecific host modulation of parasite‐induced CI is predicted to influence the evolutionary trajectory of host‐parasite interactions and, by suppressing CI strength, the long‐term effectiveness of CI‐based pest management (Turelli 1994; Ross et al. 2021, 2019). Here, we identified strong host modulation by the spider mite *T. urticae* of *Wolbachia*‐mediated CI. We collected convincing evidence that a nuclear modifier system, or systems, segregates in *T. urticae* that strongly modulates CI strength when carried by *Wolbachia*‐infected males. The lowest CI levels were observed for infected Stt‐*w* and Temp‐*w* males (CI strength dropped as low as ∼15% CI_corr_). If host modulation of CI evolved adaptively in *T. urticae*, the observed variation in CI strength would be caused by varying degrees of host suppression. Complete (or near‐complete) CI was only observed in the genetic crosses with infected Beis‐*w* males, suggesting that the Beis genotype might lack male suppressors of CI. Despite the ubiquity of CI‐inducing parasites, evidence for intraspecific host suppression of CI strength is rarely reported. Genetic work on *Drosophila* and *Nasonia* has gathered some evidence of modest intraspecific host modulation of CI strength (Perrot‐Minnot and Werren 1999; Reynolds and Hoffmann 2002; Cooper et al. 2017). However, these results remain largely inconclusive due to a lack of control for (asymmetrical) nuclear‐ or mitochondrial‐associated incompatibilities and line homozygosity. In *Drosophila simulans*, *Aedes aegypti*, and *Culex pipiens*, no intraspecific genetic variation has (yet) been observed that modulates CI strength despite intensive sampling efforts (Hoffmann and Turelli 1988; Duron et al. 2006; Atyame et al. 2011; Carrington et al. 2011; Ross et al. 2021). These findings are in sharp contrast with our study, where we uncovered signs of host suppression in four out of five near‐isogenic lines. The maternal transmission of *Wolbachia* in tetranychid mites is sensitive to increased temperatures and is likely to be imperfect under natural conditions, preventing *Wolbachia* from reaching fixation and resulting in persistent expression of CI (Breeuwer and Jacobs 1996; Van Opijnen and Breeuwer 1999; Zélé et al. 2018). Our data therefore support models that predict the evolution of host modifiers in systems with strong CI and imperfect maternal transmission (Turelli 1994; Engelstädter and Hurst 2009). In contrast to CI, host nuclear modifiers that act against male killing, a less common parasite‐induced manipulation, have been described in a range of arthropod species (Hornett et al. 2006; Engelstädter and Hurst 2009; Kageyama et al. 2009). CI and male killing exert different selection pressures on the arthropod host (Engelstädter and Hurst 2009). Male killing reduces the fitness of infected females that transmit the parasite, whereas CI has deleterious effects on uninfected females. Moreover, in contrast to male killing, the fitness cost of CI is ablated in populations that are fixed for the reproductive parasite (Turelli 1994; Engelstädter and Hurst 2009). These divergent fitness penalties could explain the observed discrepancy in host modulation.

The mechanism by which infected males are conditionally (partially) sterilized by *Wolbachia* is not well understood (Hurst 1991; Beckmann et al. 2019a; Shropshire et al. 2020), limiting our ability to unravel the mechanistic basis of host modulation. The host can develop toxicodynamic resistance to CI by changes in the target sites of *Wolbachia* Cif proteins (coined as the defensive model in Shropshire et al. 2020). As *Wolbachia* density has been observed to positively covary with CI strength and maternal transmission efficiency (Breeuwer and Werren 1993; Shropshire et al. 2020), CI can be overcome by genetic variants in the host that dysregulate *Wolbachia* density, a mechanism that can be viewed as toxicokinetic resistance (coined as the offensive model in Shropshire et al. 2020). In *Nasonia* wasps, the host genetics of infected females contribute to variation in the maternal transmission of *Wolbachia* to the progeny (Funkhouser‐Jones et al. 2018). In our study, maternal transmission of *Wolbachia* appeared complete in all infected genetic backgrounds, including the Stt and Temp genotypes that exhibited the lowest levels of CI. The interaction of the male modifier systems with the uninfected female genotype was an important determinant for CI modulation, suggesting that variation in *Wolbachia* density in infected males does not (fully) explain CI strength variation across our diallel cross design. In *Wolbachia*‐infected wasps and fruit flies, CI strength is coupled with *Wolbachia cif* gene expression (LePage et al. 2017; Nasehi et al. 2021), and host modulation of *cif* transcription could underlie the variation in CI strength observed in our study. *Cif* genes are divided into a minimum of five phylogenetic clades, and the encoded proteins exhibit extensive variation in domain structure (Martinez et al. 2020). Although all CifB proteins have a dimer of PD‐(D/E)XK nuclease domains, the diversity of additional functional domains indicates that CI may be manifested by different biochemical mechanisms across different *Wolbachia* variants, a hypothesis that finds some support in previous work (Beckmann et al. 2019a; Martinez et al. 2020; Shropshire et al. 2020). Unfortunately, the *cif* repertoire of *Wolbachia* that infect *T. urticae* has not been identified (Zélé et al. 2020)*. Wolbachia* infection of *Tetranychus* mites is characterized by an apparent high strain diversity (Zhang et al. 2013), raising the question of how the host modifier systems of our mite panel would interact with *Wolbachia* variants that carry divergent *cif* repertoires. Although speculative, the high variability of CI strength (and phenotype) in *T. urticae* populations across the globe could be (partially) caused by different male modifiers that segregate at various frequencies (Perrot‐Minnot et al. 2002; Gotoh et al. 2007; Zélé et al. 2020). In *Drosophila teissieri*, genetic crosses suggest that CI strength could be determined by an interaction between the *Wolbachia* variant and host genotype, but formal evidence awaits (Cooper et al. 2017). Further work is required to fully understand the mechanisms of male modulation of CI in our *T. urticae* genotypes.

We also gathered evidence of strong intraspecific female modulation of the CI phenotype when paired with the Scp male genotype. In the wasp genus *Nasonia*, differences in CI phenotype across species are also (partially) attributed to female modulation (Bordenstein et al. 2003). Consistent with the *Nasonia* system, the maternal genetic effect that contributes to MD‐CI in LonX‐c is recessive (Bordenstein et al. 2003). Although the results of the backcross experiments appear consistent with polygenic Mendelian inheritance, the number of loci involved, their additivity, and effect size remain unresolved. Multiple mechanisms can underpin female modulation of MD‐CI. During embryogenesis, the maternal genetic effect could contribute to the complete elimination of paternal chromosomes, giving rise to viable haploid male offspring. Beckmann *et al*. revealed that a particular type of *Wolbachia* CifB interacts with the maternally deposited proteins karyopherin‐α and P32 and identified protamine‐histone exchange and nuclear‐protein import as target pathways (Beckmann et al. 2019b). As nuclear transport has previously been associated with genetic conflict (Tang and Presgraves 2009), it is tempting to speculate that these pathways underpin the observed maternal genetic effect of this study. Alternatively, the maternal genetic effect could result in (partial) fertilization failure by physiological changes within the female reproductive tissue. Future experiments are needed to uncover the genetic architecture of female modulation of the CI phenotype.

To conclude, we identified mechanisms of intraspecific host modulation that control CI strength by male modifier systems and modify the CI phenotype by maternal genetic effects. As both mechanisms interact with the genotype of the mating partner, we show that different complex genetic architectures underlie intraspecific host modulation of parasite‐induced CI.

## AUTHOR CONTRIBUTIONS

N.W. conceived and designed the experiments. N.W. and F.M. performed the experiments. N.W., F.M., and D.B. analyzed the data. N.W. wrote the manuscript with input from F.M. and D.B.

## CONFLICT OF INTEREST

The authors declare no conflicts of interest.

## Supporting information

supplementary material

**Table S1**. Origins of the five *Tetranychus* genotypes.
**Table S2**. PCR primers and annealing temperatures.
**Table S3**. *Wolbachia* maternal transmission in the infected near‐isogenic lines.
**Table S4**. Summary of replication and egg numbers for the different cross types.

**Figure S1**. Trace data of the *Wolbachia* *wsp* locus confirms that a single *Wolbachia* variant infects the near‐isogenic mite lines.
**Figure S2**. Observed and estimated proportion of adult females over the total number of eggs across the intraspecific cross types.
**Figure S3**. Model comparisons for the analysis of intraspecific CI strength variation.
**Figure S4**. Estimated finite‐population standard deviation (SD) of coefficients of the levels of model (3) for intraspecific CI strength to quantify the relative impact of the explanatory variables.
**Figure S5**. Levels of interaction with the male infection state of intraspecific CI strength variation on the log‐odds scale.
**Figure S6**. Infected Beis‐*w* and Scp‐*w* females rescue CI.
**Figure S7**. Observed and estimated male proportion over total number of eggs across the intraspecific cross types.
**Figure S8**. Observed and estimated proportion of eggs that failed to generate adult mites over the total number of eggs that did not generate adult males (FM) across the intraspecific cross types.
**Figure S9**. Model comparisons for the analysis of the CI phenotypes in the intraspecific crosses.
**Figure S10**. Estimated finite‐population standard deviation (SD) of coefficients of the levels of model (9) and (14) for the CI phenotypes to quantify the relative impact of the explanatory variables.
**Figure S11**. Correlation of CIcorr and MDcorr (A) and FMcorr (B) across the intraspecific cross types.
**Figure S12**. CI strength remains stable for heterozygous F1 females and recombinant F2 females.
**Figure S13**. Estimated fraction of high‐MD phenotypes quantified by the weight of high‐MD distribution of the mixed model for recombinant F2 females.

Supplementary materials and methods

## Data Availability

The multilocus sequence type data and the sequence data of the *wsp* locus of the CI‐inducing *Wolbachia* variant are accessible at NCBI (OK669102‐OK669106 and OM459770). The *COI* sequence data of the Scp‐*w* line are accessible at NCBI (OL333562). Raw count data and scripts are publicly available (https://doi.org/10.5061/dryad.b2rbnzshz and https://github.com/fremorti/Host_modulation_of_parasite‐induced_CI).
