# Supplementary material for: Interacting host modifier systems control Wolbachia‐induced cytoplasmic incompatibility in a haplodiploid mite
Source: Evol Lett. 2022 May 11;6(3):255–65. doi: 10.1002/evl3.282 (PMC9233175; doi:10.1002/evl3.282)
Supplement: Supplementary file 2 — Table S1. Origins of the five Tetranychus genotypes. Table S2. PCR primers and annealing temperatures. Table S3. Wolbachia maternal transmission in the infected near‐isogenic lines. Table S4. Summary of replication and egg numbers for the different cross types. [file EVL3-6-255-s002.pdf]

Interacting host modifier systems control *Wolbachia*-induced cytoplasmic incompatibility in a haplodiploid mite

Supplementary tables

Table S1. Origins of the five *Tetranychus* genotypes

Species	Name	Host plant	Sampling site and date
T. urticae	Beis	Solomon's seal	Brugge, Belgium (2020)
	LonX	Apple	Ontario, Canada (2000's)
	Scp-*w*	Cucumber	Bredene, Belgium (2020)
	Stt	Rose	Gent, Belgium (2020)
	Temp	Frangipani	De Haan, Belgium (2020)

Table S2. PCR primers and annealing temperatures

Species	Gene	Primer name	Primer sequence (5'-3')	Ann. temp
Tetranychus	*COI*	LCO1490	GGTCAACAAATCATAAAGATATTGG	48 °C
		HCO2198	TAAACTTCAGGGTGACCAAAAAATCA	
Wolbachia	*wsp*	*wsp*_81F	TGGTCCAATAAGTGATGAAGAAAC	54 °C
		*wsp*_691R	AAAAATTAAACGCTACTCCA	
	wsp locus	*wsp*loc_F	TGAAATAGGTGTCAGAAGATCAAG	54 °C
		*wsp*loc_R	TGTGTTATTCCAGTGCTCAAAC	
	gatB	*gatB*_F1	GAKTTAAAYCGYGCAGGBGTT	54 °C
		*gatB*_R1	TGGYAAYTCRGGYAAAGATGA	
	coxA	*coxA*_F1	TTGGRGCRATYAACTTTATAG	54 °C
		*coxA*_R1	CTAAAGACTTTKACRCCAGT	
	hcpA	*hcpA*_F1	GAAATARCAGTTGCTGCAAA	54 °C
		*hcpA*_R1	GAAAGTYRAGCAAGYTCTG	
	ftsZ	*ftsZ*_F1	ATYATGGARCATATAAARGATAG	54 °C
		*ftsZ*_R1	TCRAGYAATGGATTRGATAT	
	fbpA	*fbpA*_F1	GCTGCTCCRCTTGGYWTGAT	55 °C
		*fbpA*_R1	CCRCCAGARAAAAYYACTATTC	
Rickettsia	*gtlA*	RICS741F	CATCCGGAGCTAATGGTTTTGC	52 °C
		RCIT1197R	CATTTCTTTCCATTGTGCCATC	
Cardinium	16S rRNA	ChF	TACTGTAAGAATAAGCACCGGC	52 °C
		ChR	GTGGATCACTTAACGCTTTCG	
Spiroplasma	spacer region	SpitsJ04_F	GCCAGAAGTCAGTGTCCTAACCG	52 °C
		SpitsN55_R	ATTCCAAGGCATCCACCATACG	

PCR assays were performed using DreamTaq DNA Polymerase (Life Technologies Europe B.V.) in a 50 µl reaction mixture. Thirty cycles were run for all PCR reactions. *Wolbachia* infection was tested using the primers that amplify a fragment of *wsp*. The diagnostic PCR assays for the detection of reproductive manipulators have been extensively tested on spider mites and other arthropods in previous studies. A *Wolbachia*-infected *Myrmica scabrinodis* worker and laboratory populations of *T. urticae* and *Bryobia* species that were infected with *Wolbachia*, *Rickettsia*, *Cardinium*, and *Spiroplasma* were used as positive controls. Using genome sequence data, the *wsp*loc primer pair is designed to amplify a ~1,000 bp fragment of *Wolbachia* of *Chrysomya megacephala* (CP021120.1), *Aedes albopictus* (CP031221.1), *Bemisia tabaci* (CP016430.1), *Spodoptera picta* (CP067976.1), *Diaphorina citri* (CP051608.1), *Culex quinquefasciatus* (AM999887.1), *Corcyra cephalonica* (CP087954.1), *Nasonia vitripennis* (*w*VitB, NZ_GL883634.1), *Leptopilina clavipes* (NZ_QJHA01000035.1), and *Anopheles demeilloni* (CP084694.1). The genomic fragment exhibited 204 polymorphic sites across the focal *Wolbachia* and reference isolates, including multiple indels.

Table S3. *Wolbachia* maternal transmission in the infected near-isogenic lines

**Name**	♀	♂
Beis-*w*	35	13
LonX-*w*	29	10
Scp-*w*	31	12
Stt-*w*	30	13
Temp-*w*	31	14

All adult females and males were infected with *Wolbachia,* indicating complete maternal transmission in the five infected near-isogenic lines.

Table S4. Summary of replication and egg numbers for the different cross types

		Infected males (-*w*)			Cured males (-c)		
Cross	Cross type (♀ x ♂)	N	Egg number	SE	N	Egg number	SE
**Intraspecific**	Beis-c x Beis	11	61.45455	6.950950	10	65.00000	6.487595
	Beis-c x LonX	10	63.70000	5.368737	10	57.30000	7.892401
	Beis-c x Scp	9	66.11111	5.898315	8	80.75000	5.270369
	Beis-c x Stt	11	90.45455	5.871911	10	73.10000	8.459643
	Beis-c x Temp	11	76.90909	10.237591	10	83.00000	10.164207
	LonX-c x Beis	10	110.60000	7.917912	10	115.80000	13.157001
	LonX-c x LonX	10	56.00000	5.771578	10	70.60000	6.888315
	LonX-c x Scp	11	85.09091	6.204770	9	103.88889	9.419903
	LonX-c x Stt	11	110.36364	12.220157	11	130.45455	14.180245
	LonX-c x Temp	11	75.18182	5.802536	10	63.10000	6.089061
	Scp-c x Beis	11	81.00000	8.096015	10	73.00000	7.215724
	Scp-c x LonX	11	59.27273	7.142817	11	55.54545	3.303892
	Scp-c x Scp	11	67.00000	4.312772	8	62.00000	6.472469
	Scp-c x Stt	11	106.54545	11.234981	11	97.09091	9.589250
	Scp-c x Temp	10	55.80000	4.701773	10	51.40000	5.566168
	Stt-c x Beis	11	87.45455	6.145750	10	84.20000	6.432211
	Stt-c x LonX	11	104.54545	11.375703	9	94.88889	10.454192
	Stt-c x Scp	10	76.30000	6.331666	10	85.90000	7.227186
	Stt-c x Stt	11	80.45455	8.157945	10	82.80000	7.853945
	Stt-c x Temp	11	81.09091	5.801966	10	76.80000	6.018121
	Temp-c x Beis	11	101.54545	9.004407	10	86.50000	7.019418
	Temp-c x LonX	8	66.62500	15.260754	8	74.12500	12.214508
	Temp-c x Scp	8	64.12500	4.958533	8	57.12500	6.833315
	Temp-c x Stt	11	163.36364	9.610858	11	130.00000	8.918826
	Temp-c x Temp	8	59.12500	9.611075	8	53.75000	3.260970
**Rescue**	Beis-*w* x Beis	5	76.80000	6.429619	5	66.40000	6.749815
	Scp-*w* x Scp	5	84.40000	3.325658	5	48.60000	3.558089

Average egg numbers and their standard error (SE) are listed. Number of replicates is indicated by N.
