# Supplementary material for: Interacting host modifier systems control Wolbachia‐induced cytoplasmic incompatibility in a haplodiploid mite
Source: Evol Lett. 2022 May 11;6(3):255–65. doi: 10.1002/evl3.282 (PMC9233175; doi:10.1002/evl3.282)
Supplement: Supplementary file 4 — Supplementary materials and methods [file EVL3-6-255-s004.pdf]

Interacting host modifier systems control *Wolbachia*-induced cytoplasmic incompatibility in a haplodiploid mite

Supplementary materials and methods

Single mite DNA extraction

Individual mites were isolated and homogenized in 21 µl of PCR buffer (10 mM Tris-HCl, 100 mM NaCl, 1 mM EDTA, 2 mg/ml of proteinase K, with pH 8). Homogenates were incubated at 37°C for 30 min. Proteinase K was inactivated by incubating the mite samples at 95°C for 10 min. Each mite sample was diluted by adding 10 µl of sterile nuclease-free water.

Curing *T. urticae* of *Wolbachia* infection

Leaf discs of 16 cm^2 were dehydrated at 60°C for 1 min and soaked in a rifampicin solution (0.025 mg/ml). A cured line was created for each *Wolbachia*-infected line by transferring 40 larvae to a rifampicin-treated leaf disc and developing on rifampicin-treated leaf discs for two successive generations. Leaf discs were replaced every three days and rifampicin solutions were refreshed daily. After maintaining the lines on detached bean leaves for three generations, antibiotic curing of *Wolbachia* infection was confirmed by diagnostic PCR assays on 25 individual adult females and a pool of 100 adult females. During the experiments, we confirmed an absence of *Wolbachia* infection for the Beis-c and Scp-c lines by diagnostic PCR assays on a pool of 50 adult females. Five months after all experiments were completed, we provided a final formal demonstration that all cured lines (Beis-c, LonX-c, Scp-c, Stt-c, and Temp-c) remained free of *Wolbachia* infection by diagnostic PCR assays on pools of ~150 adult females. Two adult females of the respective *Wolbachia*-infected sister line were consistently processed in parallel and served as positive controls for the PCR assays. PCR conditions are described in Table S2.
